# Supplementary material for: Secondary Metabolites from Aspergillus sparsus NBERC_28952 and Their Herbicidal Activities
Source: Plants (Basel). 2023 Jan 3;12(1):203. doi: 10.3390/plants12010203 (PMC9823327; doi:10.3390/plants12010203)
Supplement: Supplementary file 1 [file plants-12-00203-s001.zip › plants-2094628-supplementary.pdf]

Supplementary information for

Secondary metabolites from *Aspergillus sparsus* NBERC_28952 and their herbicidal activities

Zhaoyuan Wu[1,#], Fang Liu[1,#], Shaoyong Ke[1,], Zhigang Zhang[1,], Hongtao Hu[1], Wei Fang[1,], Shaoyujia Xiao[2],Yani Zhang[1,], Yueying Wang[1,], and Kaimei Wang[1,*]

1 Hubei Biopesticide Engineering Research Centre, Hubei Academy of Agricultural Sciences, Wuhan 430064, P. R. China
2 Department of Pharmacy, Medical College, Wuhan University of Science and Technology, Wuhan 430065, P. R. China
# These authors contributed equally to this work.
* Correspondence: kaimei.wang@nberc.com; Tel.: +86-27-59101928

Figure S1 Mass spectrum of compound **1** (determined as $C_{23}H_{30}O_5$ by HR-ESI-MS: *m/z* 387.2170, $[M+H]^+$, cald for $C_{23}H_{31}NaO_5$, 387.2166)
Figure S2 ^{1}H-NMR spectrum of compound **1**
Figure S3 ^{13}C-NMR spectrum of compound **1**
Figure S4 ^{1}H-^{13}C HSQC spectrum of compound **1**
Figure S5 ^{1}H-^{1}H COSY spectrum of compound **1**
Figure S6 ^{1}H-^{13}C HMBC spectrum of compound **1**
Figure S7 ^{1}H-^{1}H NOESY spectrum of compound **1**
Figure S8 Mass spectrum of compound **2** (determined as $C_{23}H_{30}O_5$ by HR-ESI-MS: *m/z* 387.2163, $[M+H]^+$, cald for $C_{23}H_{31}O_5$, 387.2166)
Figure S9 ^{1}H-NMR spectrum of compound **2**
Figure S10 ^{13}C-NMR spectrum of compound **2**
Figure S11 ^{1}H-^{13}C HSQC spectrum of compound **2**
Figure S12 ^{1}H-^{1}H COSY spectrum of compound **2**
Figure S13 ^{1}H-^{13}C HMBC spectrum of compound **2**
Figure S14 ^{1}H-^{1}H ROESY spectrum of compound **2**
Figure S15 Mass spectrum of compound **3** (determined as $C_{23}H_{30}O_5$ by HR-ESI-MS: *m/z* 387.2167, $[M+H]^+$, cald for $C_{23}H_{31}O_5$, 387.2166)
Figure S16 ^{1}H-NMR spectrum of compound **3**
Figure S17 ^{13}C-NMR spectrum of compound **3**
Figure S18 ^{1}H-^{13}C HSQC spectrum of compound **3**
Figure S19 ^{1}H-^{1}H COSY spectrum of compound **3**
Figure S20 ^{1}H-^{13}C HMBC spectrum of compound **3**
Figure S21 ^{1}H-^{1}H ROESY spectrum of compound **3**
Figure S22 Mass spectrum of compound **4** (determined as $C_{25}H_{34}O_6$ by HR-ESI-MS: *m/z* 453.2255, $[M+Na]^+$, cald for $C_{25}H_{34}NaO_6$, 453.2248)
Figure S23 ^{1}H-NMR spectrum of compound **4**
Figure S24 ^{13}C-NMR spectrum of compound **4**
Figure S25 ^{1}H-^{13}C HSQC spectrum of compound **4**

Figure S26 ^{1}H-^{1}H COSY spectrum of compound **4**
Figure S27 ^{1}H-^{13}C HMBC spectrum of compound **4**
Figure S28 ^{1}H-^{1}H ROESY spectrum of compound **4**
Figure S29 Mass spectrum of compound **5** (determined as $C_{22}H_{26}O_5$ by HR-ESI-MS: *m*/*z* 371.1857, $[M+Na]^+$, cald for $C_{22}H_{27}O_5$, 371.1853)
Figure S30 ^{1}H-NMR spectrum of compound **5**
Figure S31 ^{13}C-NMR spectrum of compound **5**
Figure S32 ^{1}H-^{13}C HSQC spectrum of compound **5**
Figure S33 ^{1}H-^{1}H COSY spectrum of compound **5**
Figure S34 ^{1}H-^{13}C HMBC spectrum of compound **5**

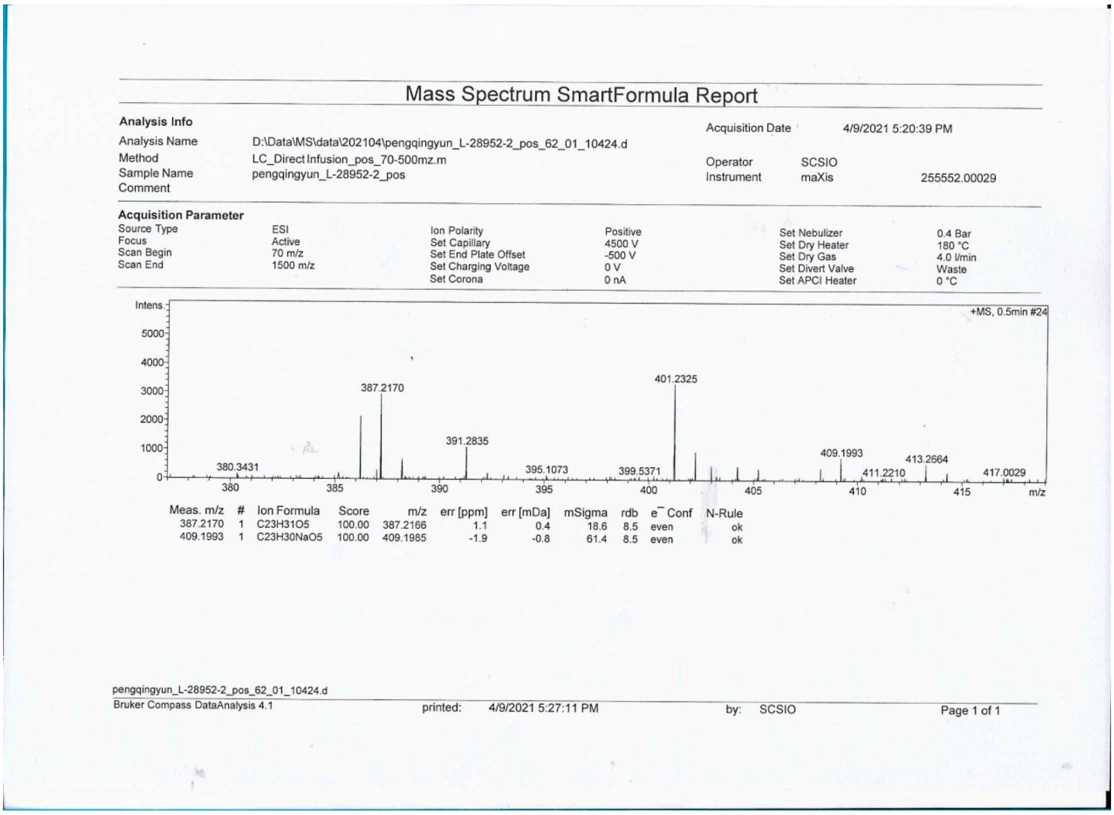

Figure S1 Mass spectrum of compound **1** (determined as $C_{23}H_{30}O_5$ by HR-ESI-MS: *m*/*z* 387.2170, $[M+H]^+$, cald for $C_{23}H_{31}NaO_5$, 387.2166)

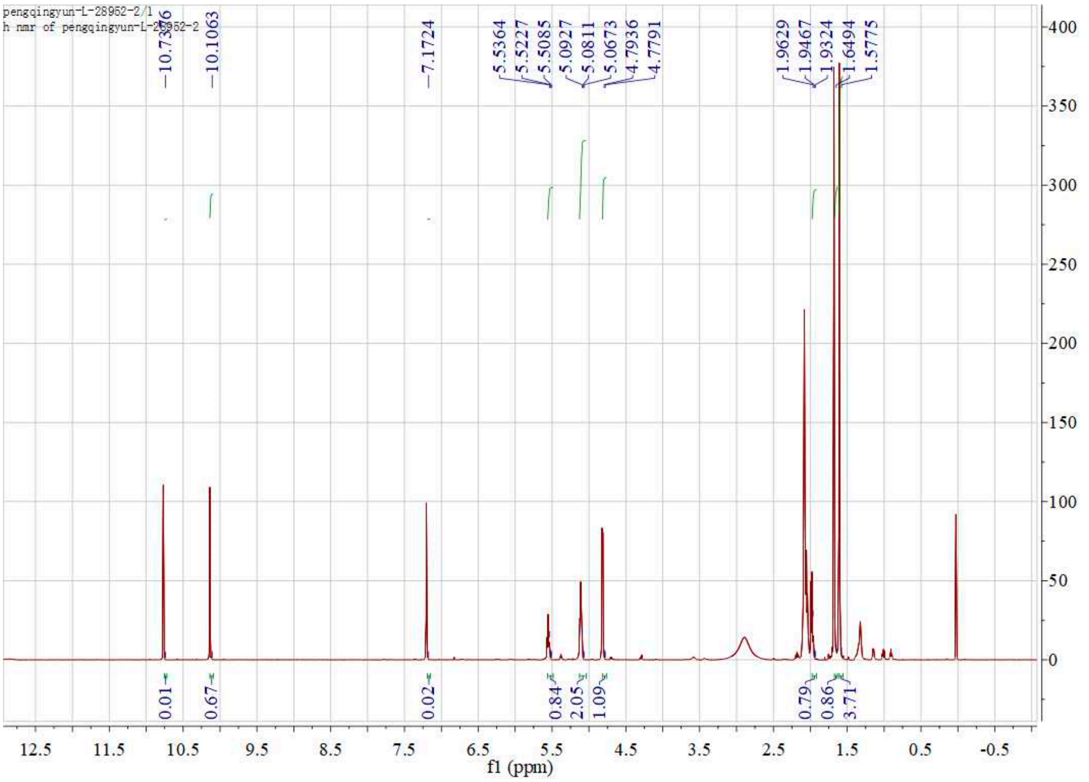

Figure S2 ^{1}H-NMR spectrum of compound **1**

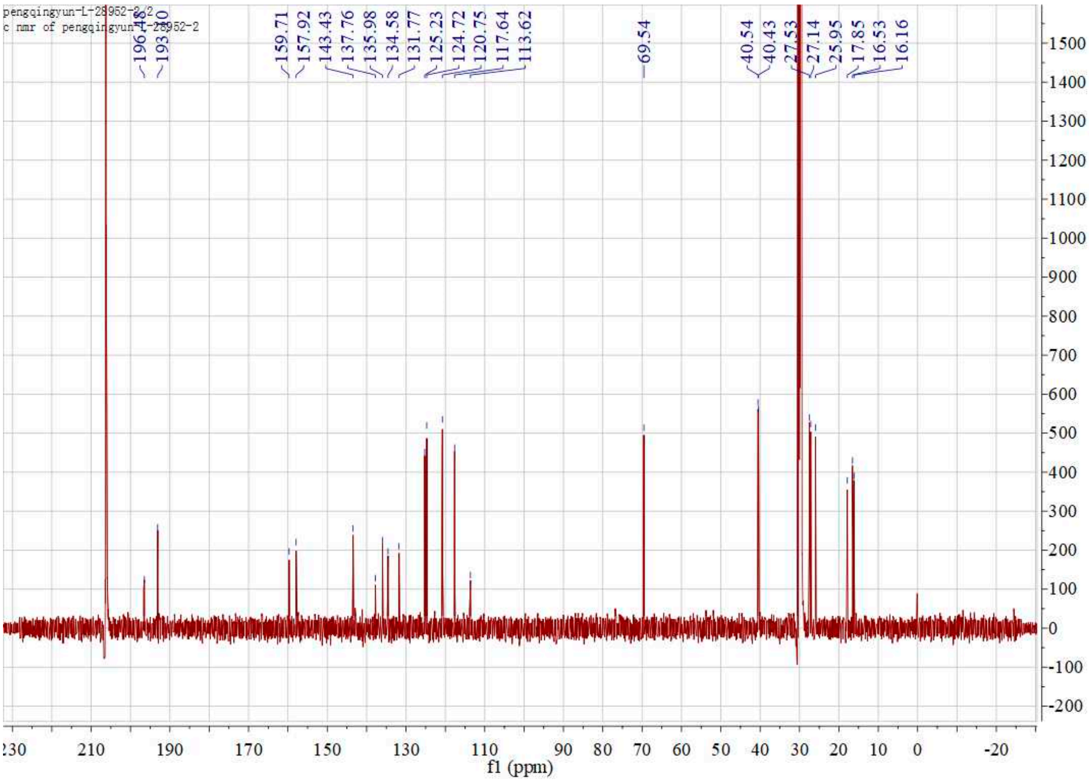

Figure S3 ^{13}C-NMR spectrum of compound **1**

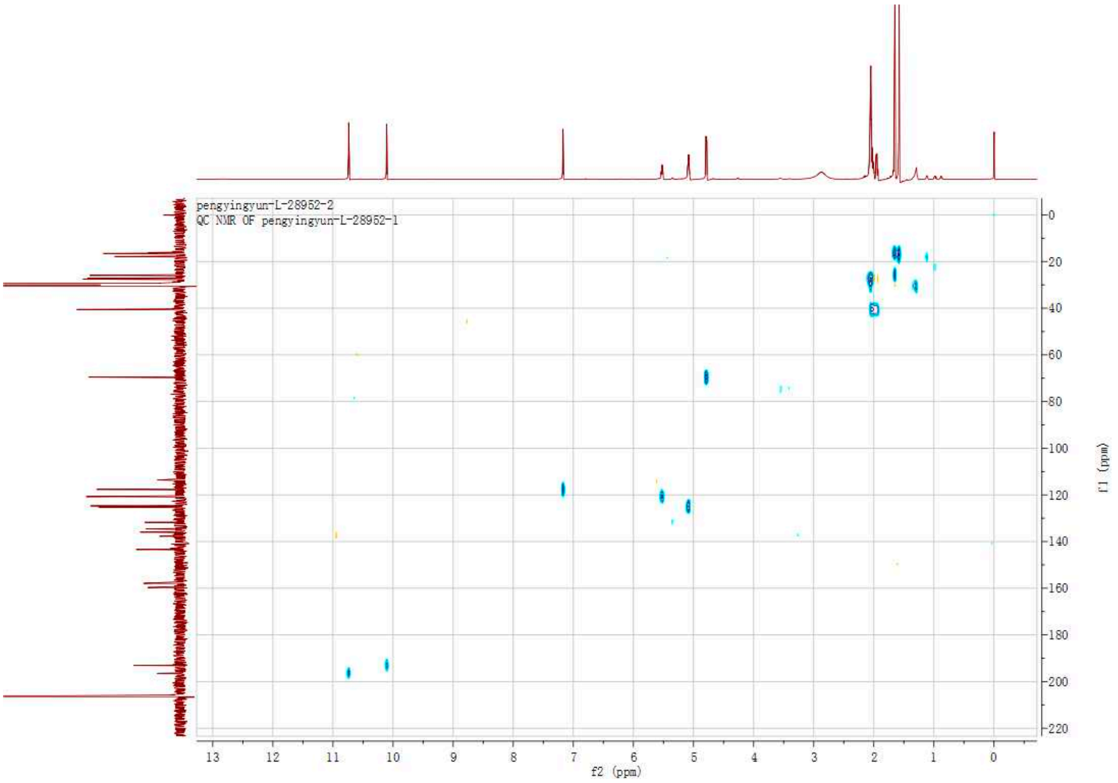

Figure S4 HSQC spectrum of compound **1**

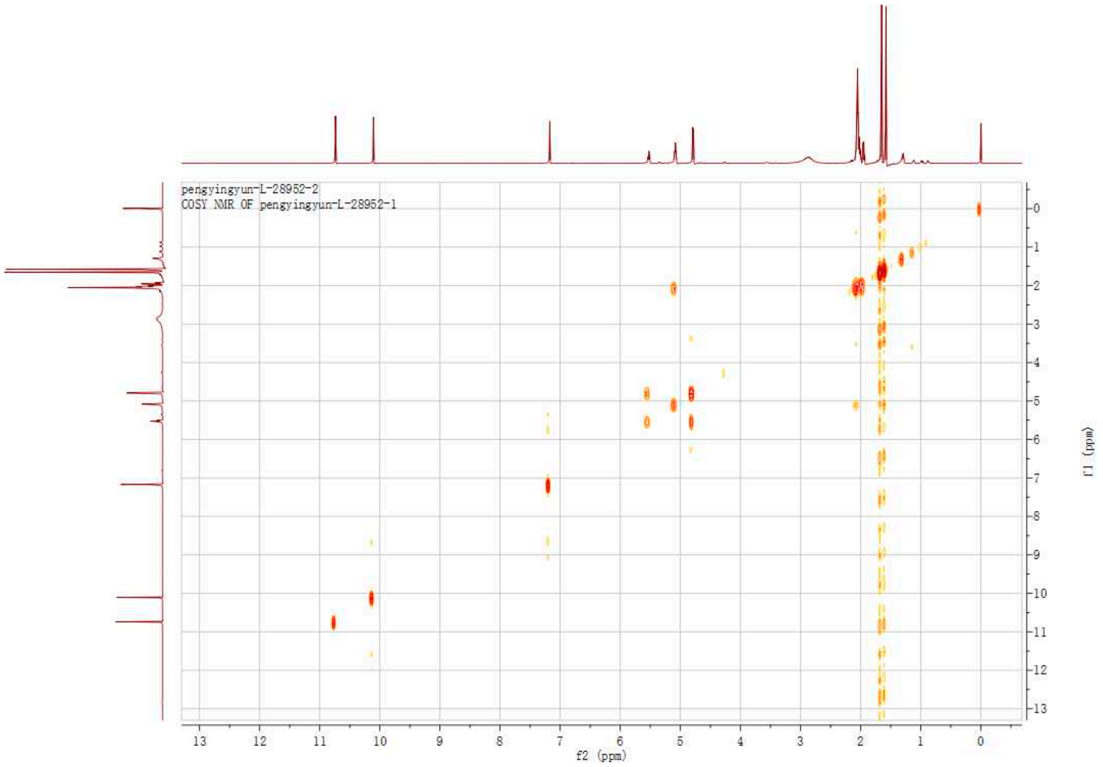

Figure S5 ^{1}H-^{1}H COSY spectrum of compound **1**

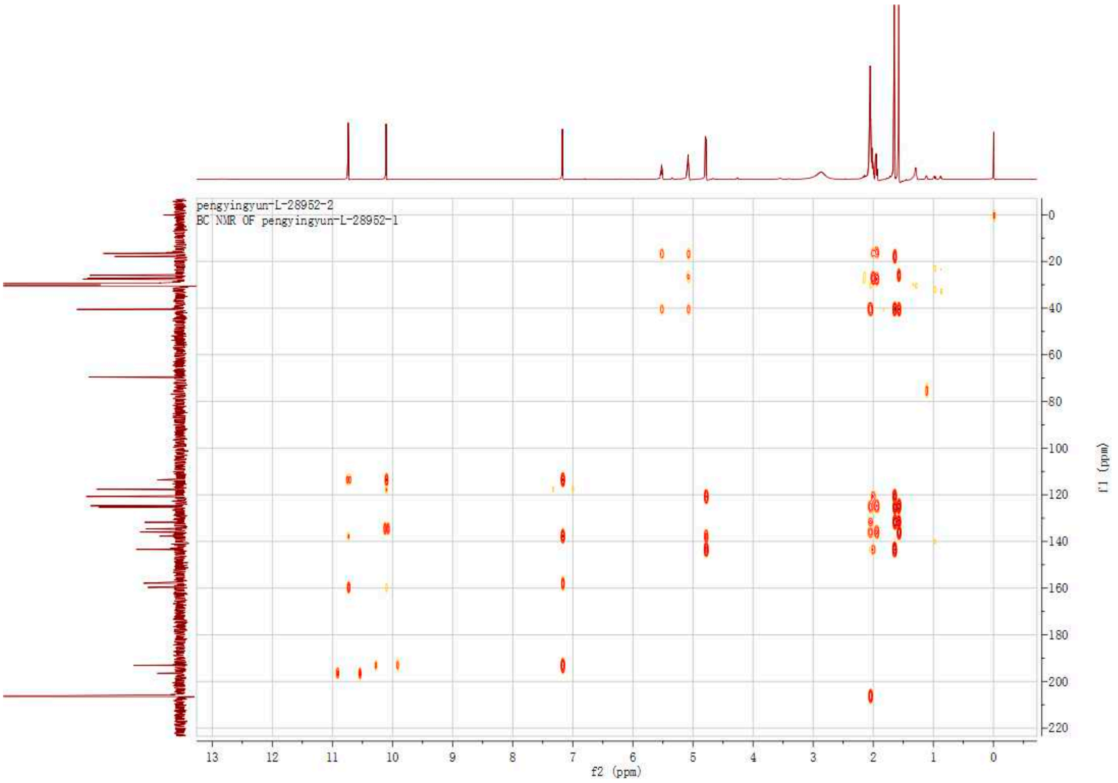

Figure S6 ^{1}H-^{13}C HMBC spectrum of compound **1**

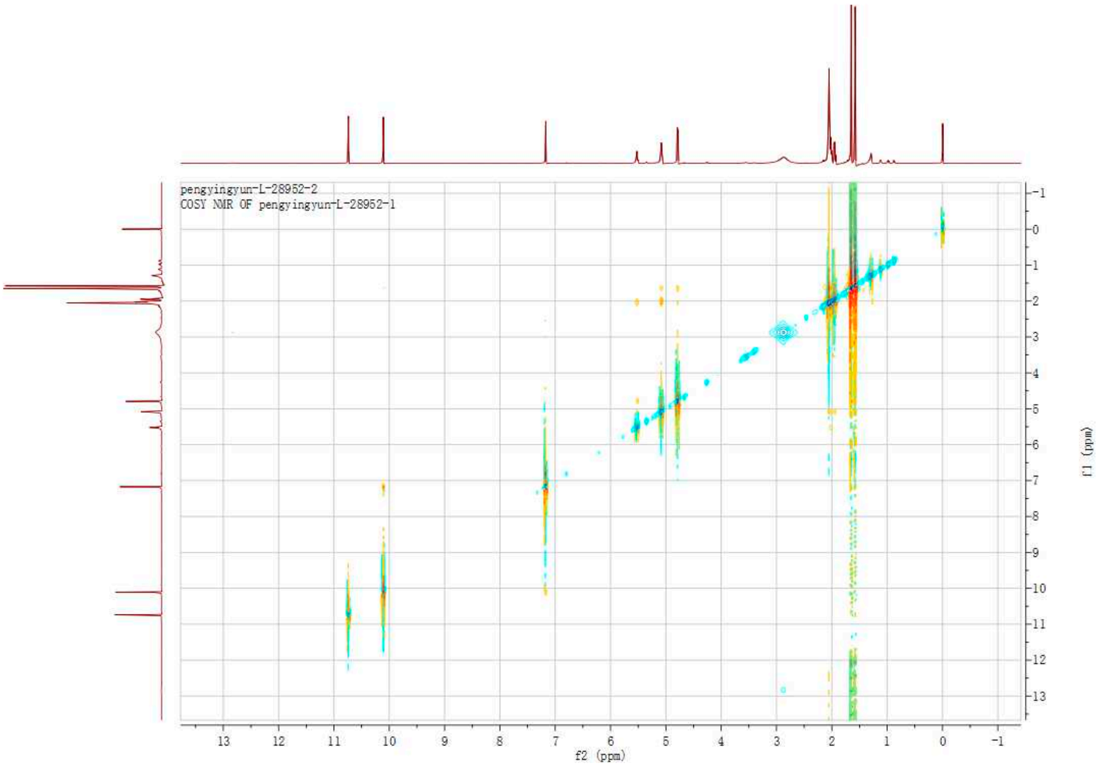

Figure S7 ^{1}H-^{1}H NOESY spectrum of compound **1**

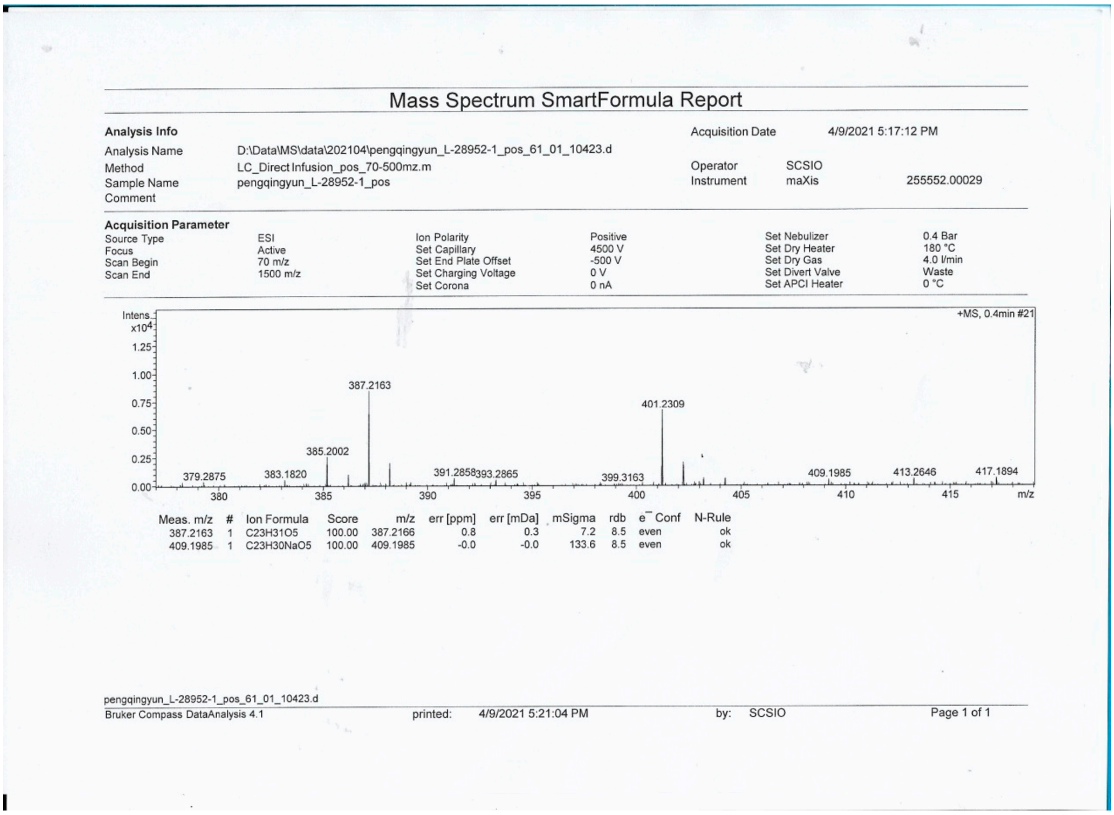

Figure S8 Mass spectrum of compound **2** (determined as $C_{23}H_{30}O_5$ by HR-ESI-MS: *m*/*z* 387.2163, $[M+H]^+$, cald for $C_{23}H_{31}O_5$, 387.2166)

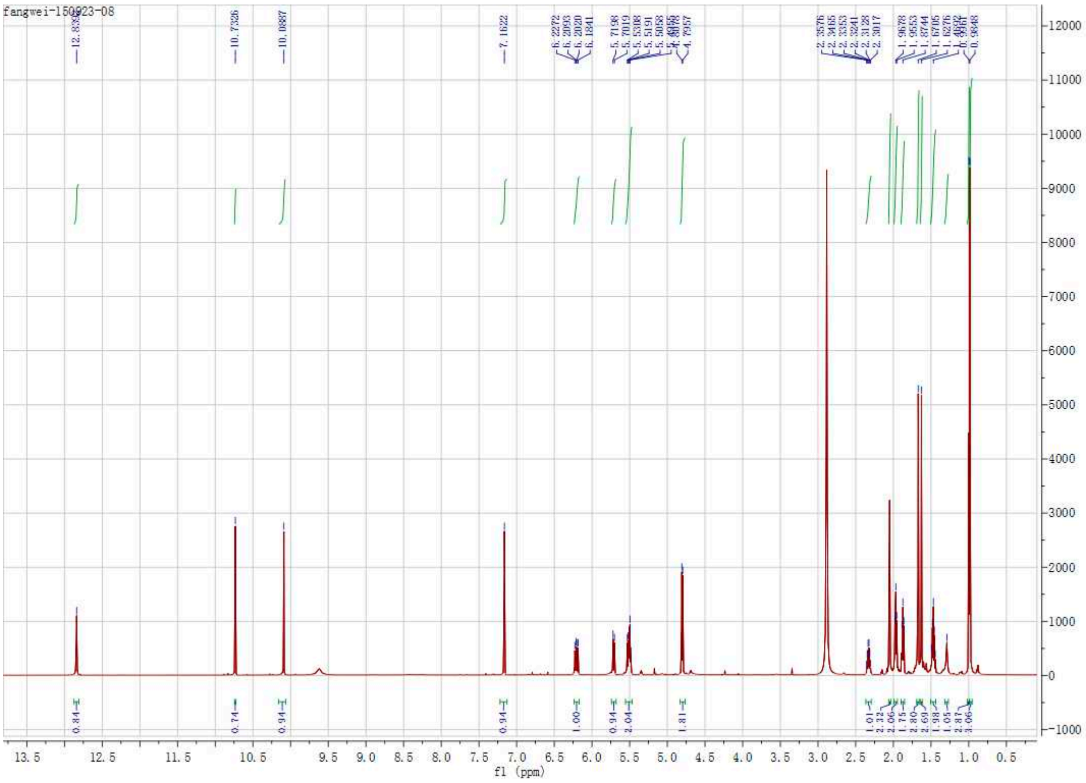

Figure S9 ^{1}H-NMR spectrum of compound **2**

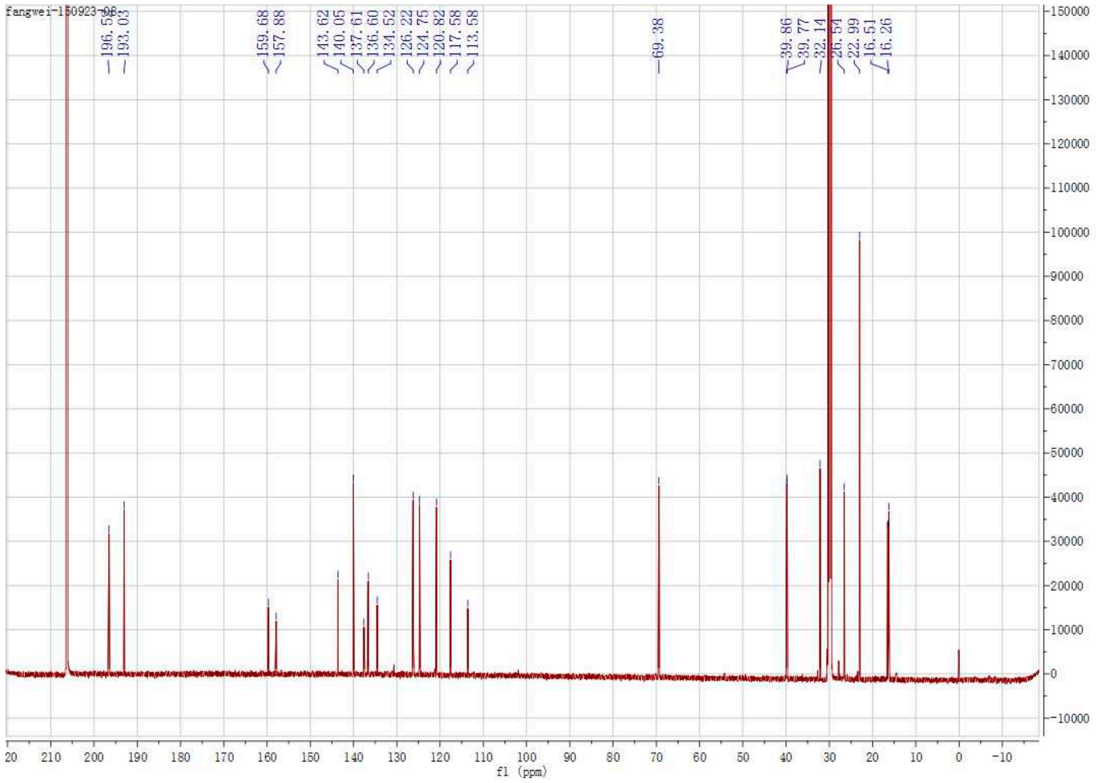

Figure S10 ^{13}C-NMR spectrum of compound **2**

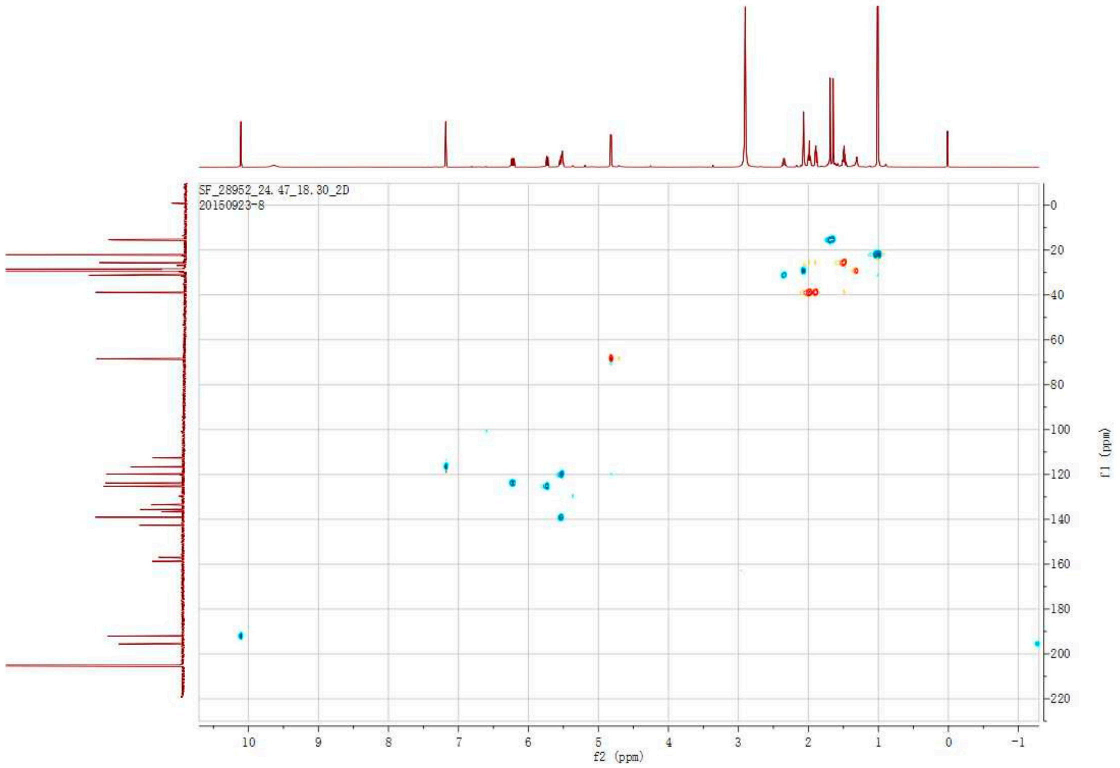

Figure S11 ^{1}H-^{13}C HSQC spectrum of compound **2**

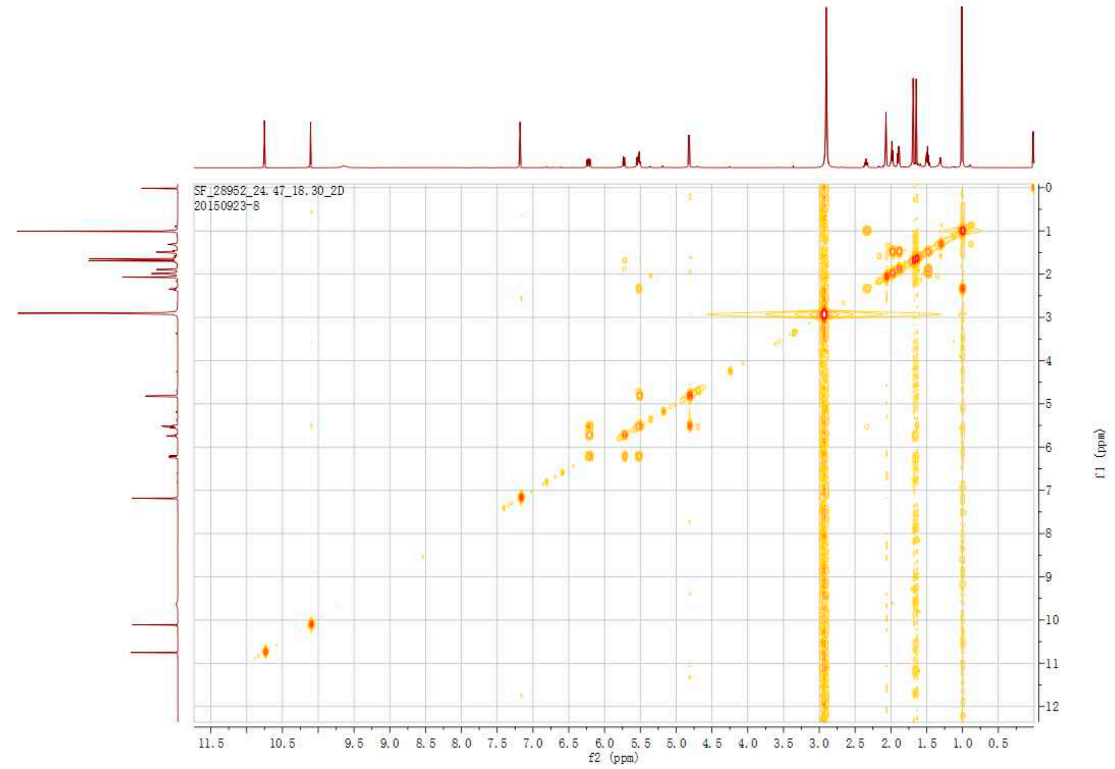

Figure S12 ^{1}H-^{1}H COSY spectrum of compound **2**

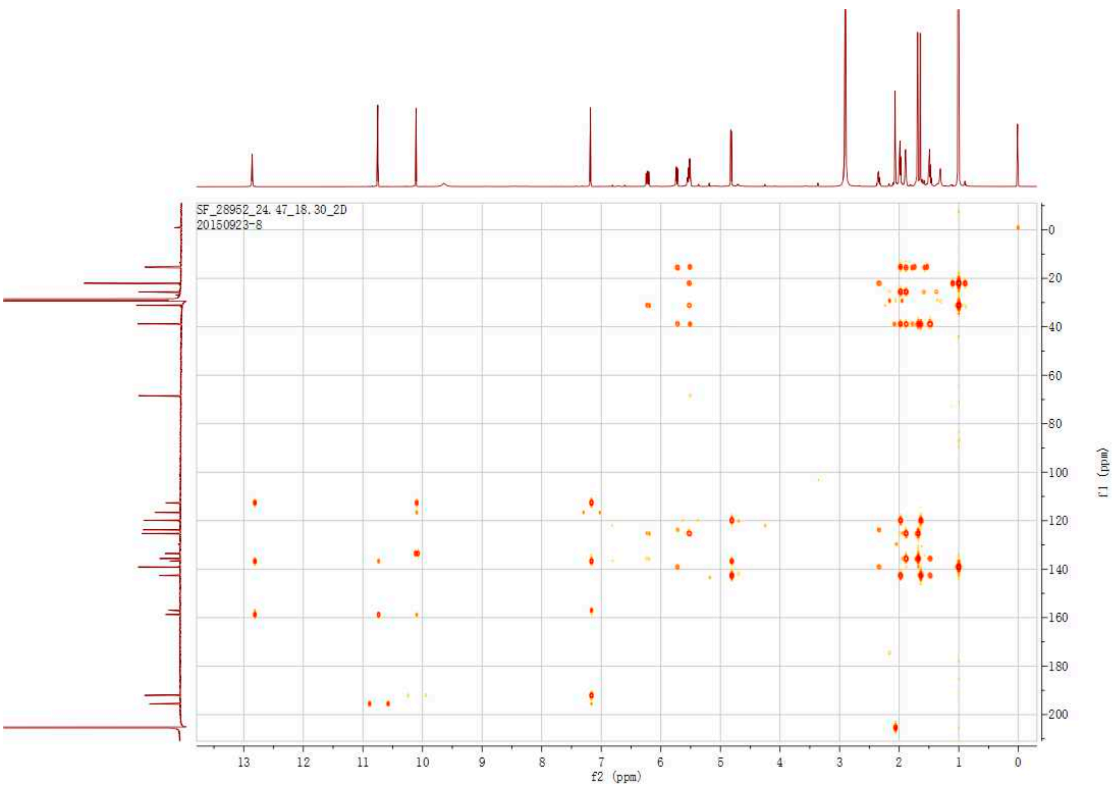

Figure S13 ^{1}H-^{13}C HMBC spectrum of compound **2**

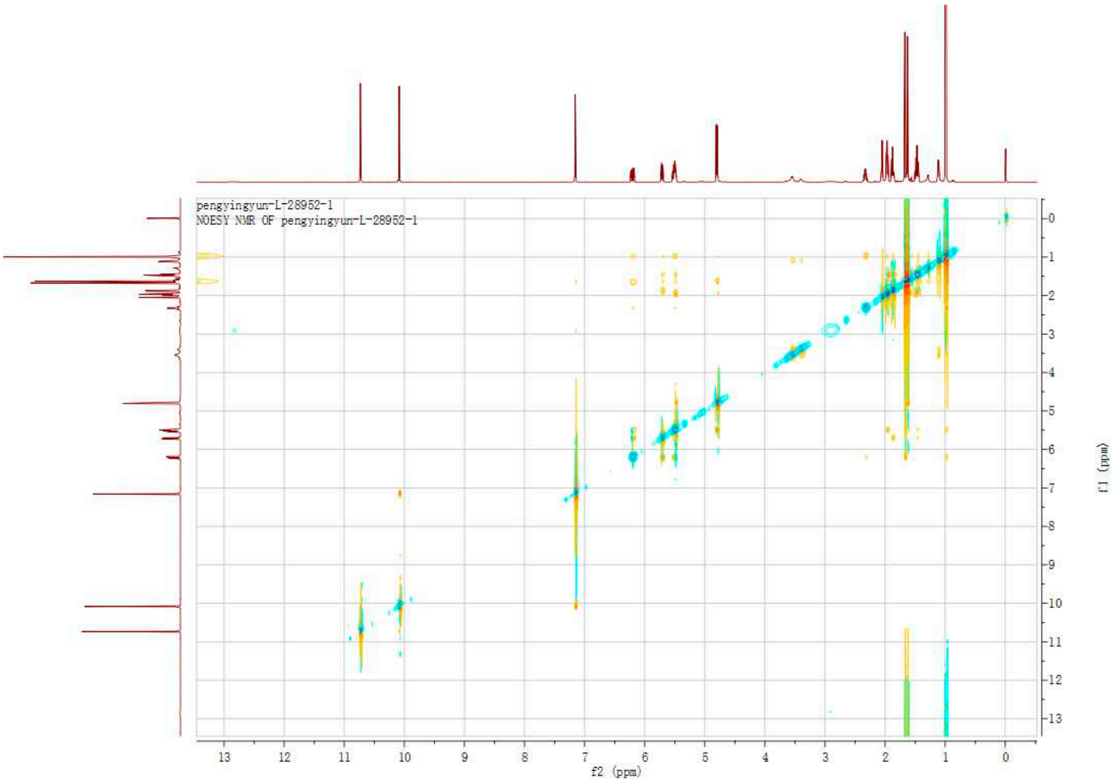

Figure S14 ^{1}H-^{1}H NOESY spectrum of compound **2**

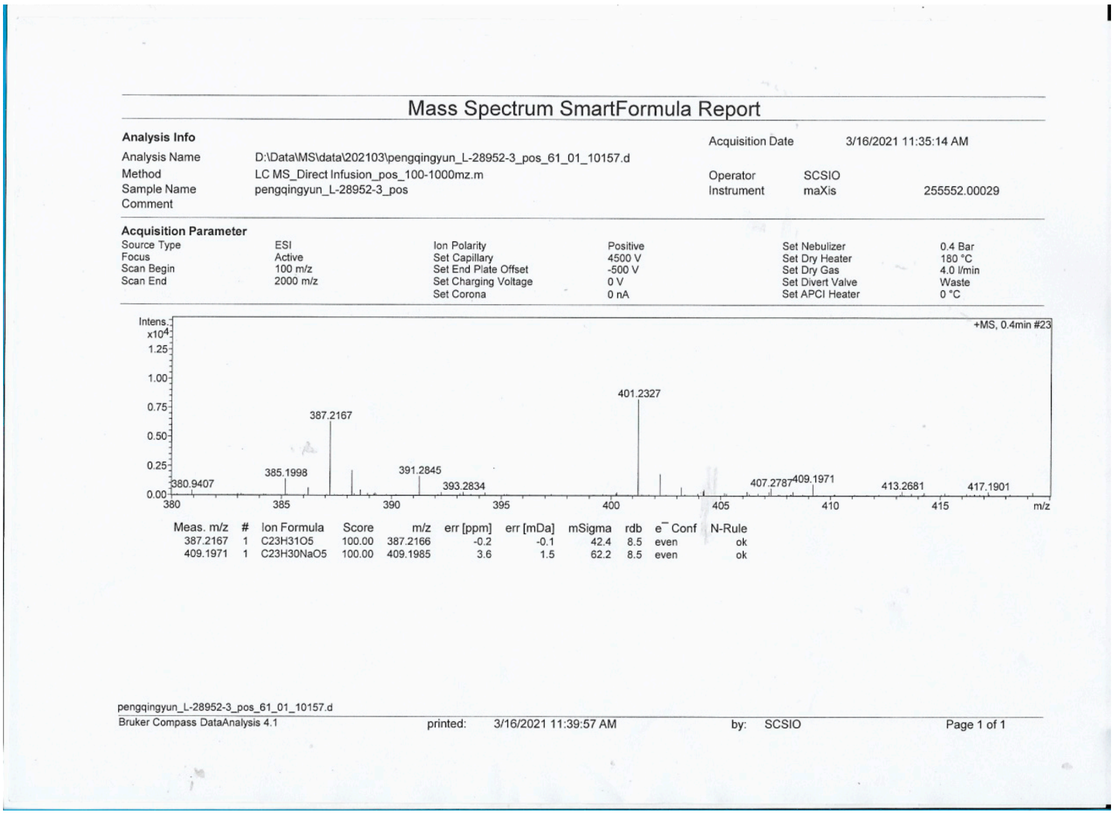

Mass Spectrum SmartFormula Report

Analysis Info

Analysis Name D:\Data\MS\data\202103\pengqingyun_L-28952-3_pos_61_01_10157.d
Method LC MS_Direct Infusion_pos_100-1000mz.m
Sample Name pengqingyun_L-28952-3_pos
Comment

Acquisition Date 3/16/2021 11:35:14 AM
Operator SCSIO
Instrument maXis 255552.00029

Acquisition Parameter

Source Type	ESI	Ion Polarity	Positive	Set Nebulizer	0.4 Bar
Focus	Active	Set Capillary	4500 V	Set Dry Heater	180 °C
Scan Begin	100 m/z	Set End Plate Offset	-500 V	Set Dry Gas	4.0 l/min
Scan End	2000 m/z	Set Charging Voltage	0 V	Set Divert Valve	Waste
		Set Corona	0 nA	Set APCI Heater	0 °C

Meas. m/z	#	Ion Formula	Score	m/z	err [ppm]	err [mDa]	mSigma	rdb	e⁻ Conf	N-Rule
387.2167	1	C23H31O5	100.00	387.2166	-0.2	-0.1	42.4	8.5	even	ok
409.1971	1	C23H30NaO5	100.00	409.1985	3.6	1.5	62.2	8.5	even	ok

pengqingyun_L-28952-3_pos_61_01_10157.d
Bruker Compass DataAnalysis 4.1 printed: 3/16/2021 11:39:57 AM by: SCSIO Page 1 of 1

Figure S15 Mass spectrum of compound **3** (determined as $C_{23}H_{30}O_5$ by HR-ESI-MS: *m/z* 387.2167, $[M+H]^+$, cald for $C_{23}H_{31}O_5$, 387.2166)

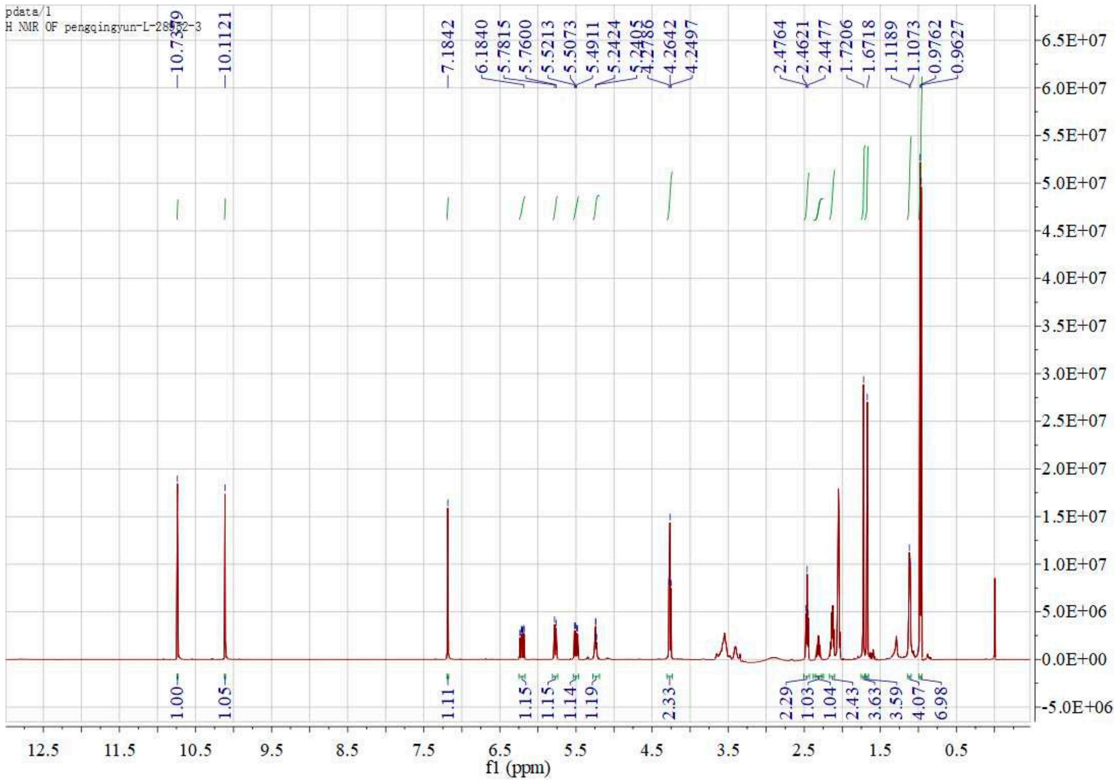

Figure S16 ^{1}H-NMR spectrum of compound **3**

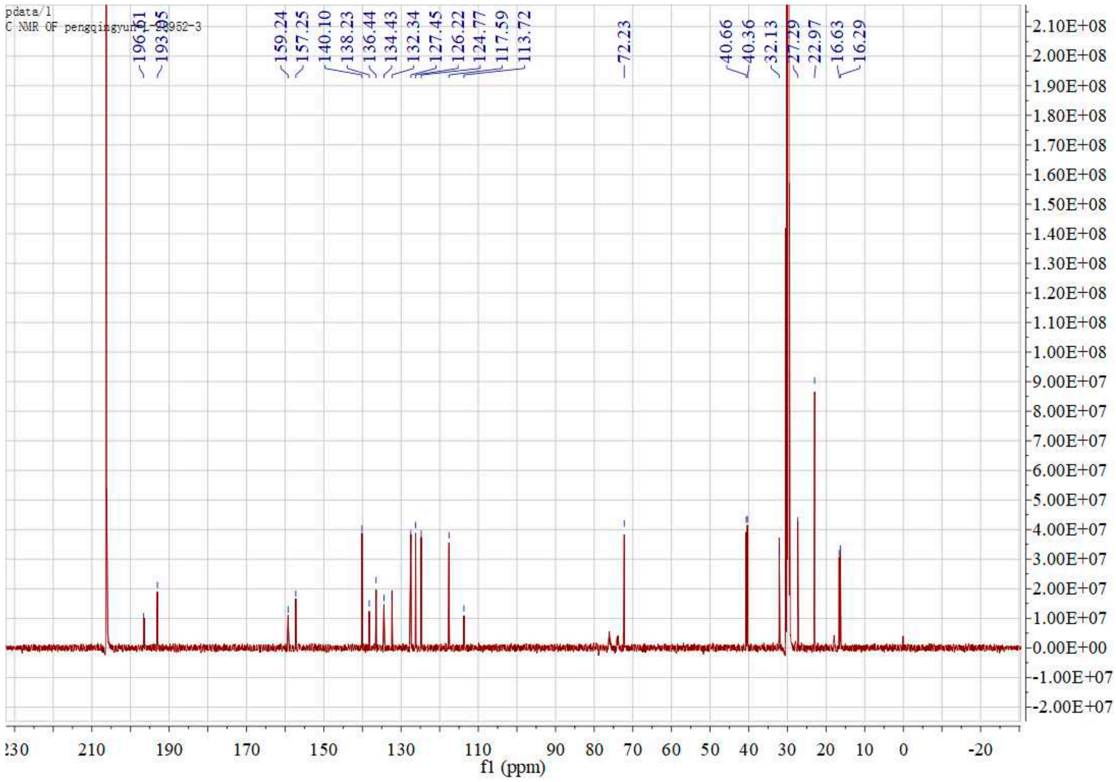

Figure S17 ^{13}C-NMR spectrum of compound **3**

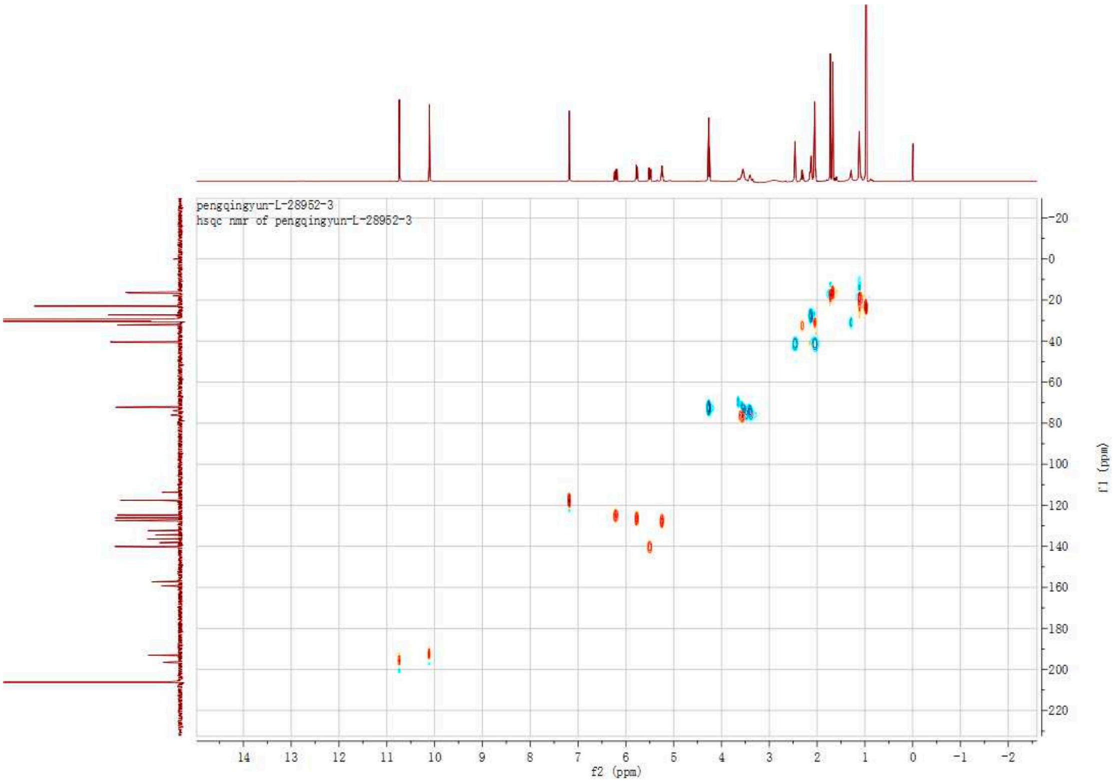

Figure S18 ^{1}H-^{13}C HSQC spectrum of compound **3**

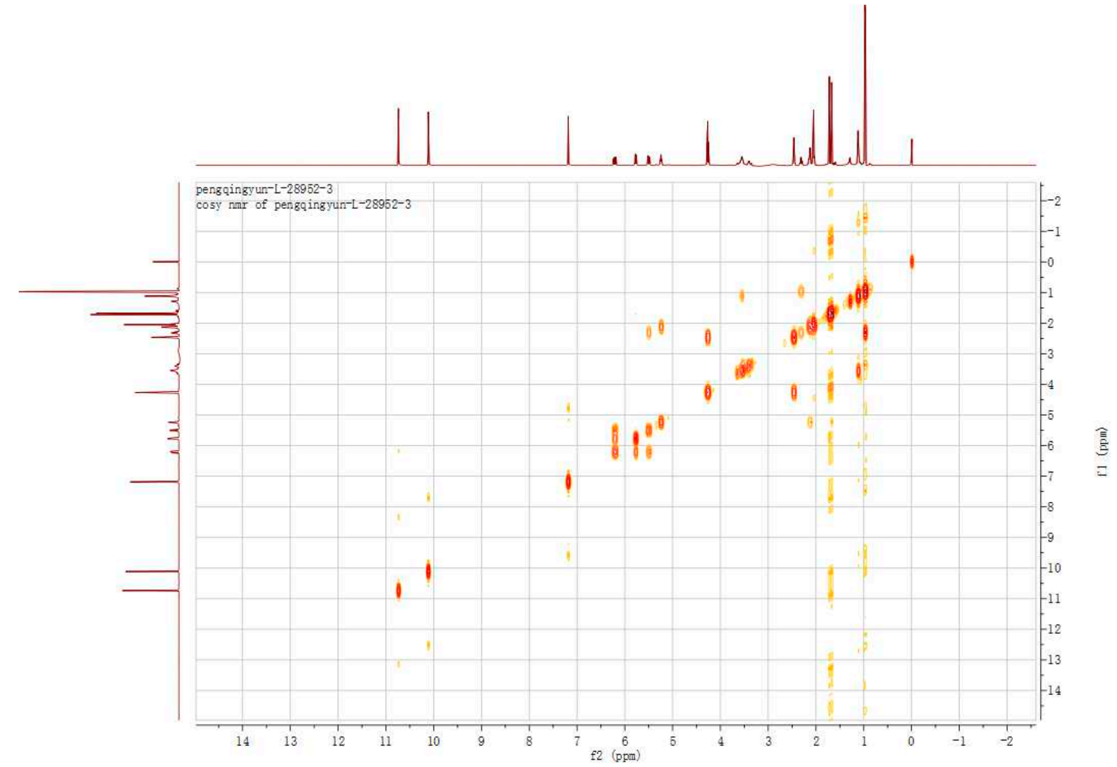

Figure S19 ^{1}H-^{1}H COSY spectrum of compound **3**

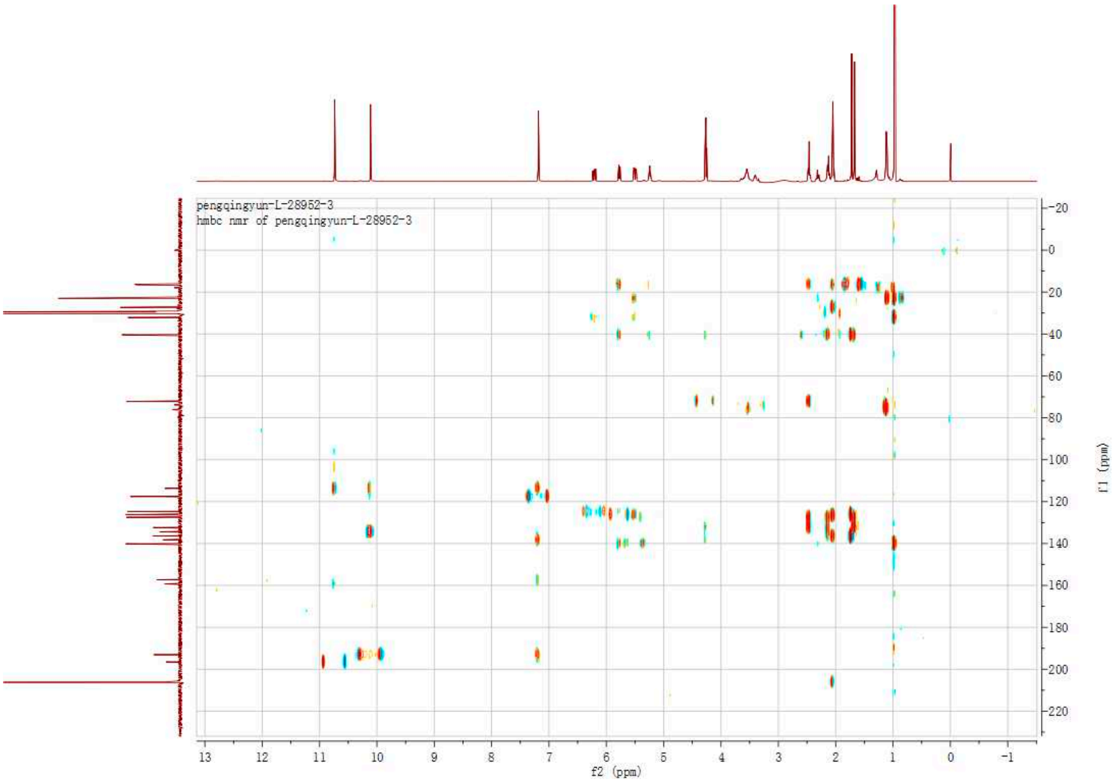

Figure S20 ^{1}H-^{13}C HMBC spectrum of compound **3**

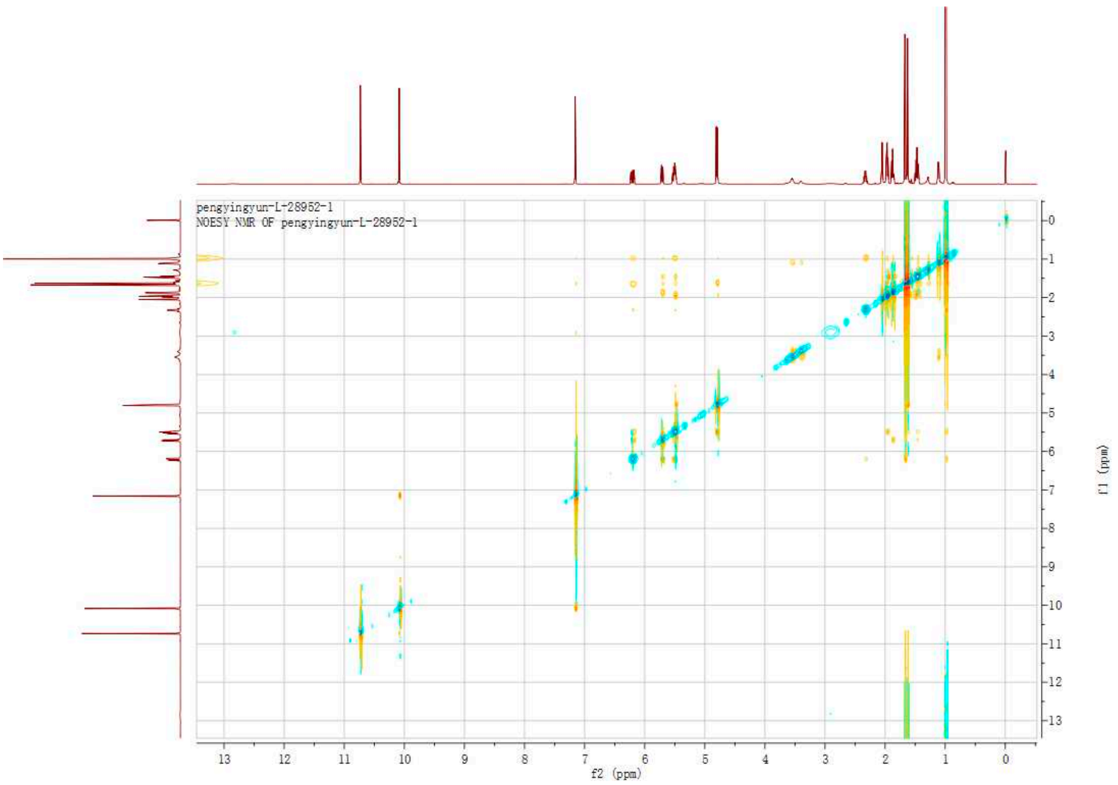

Figure S21 ^{1}H-^{1}H NOESY spectrum of compound **3**

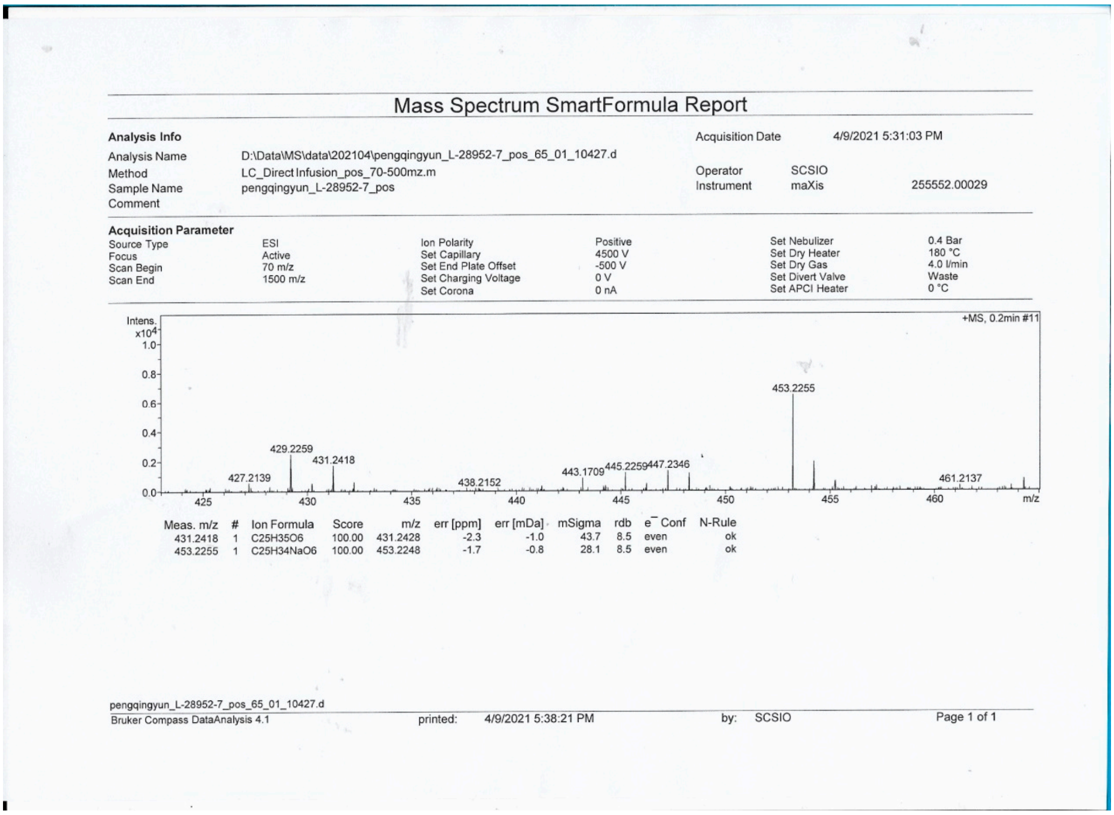

Figure S22 Mass spectrum of compound **4** (determined as $C_{25}H_{34}O_6$ by HR-ESI-MS: *m/z* 453.2255, $[M+Na]^+$, cald for $C_{25}H_{34}NaO_6$, 453.2248)

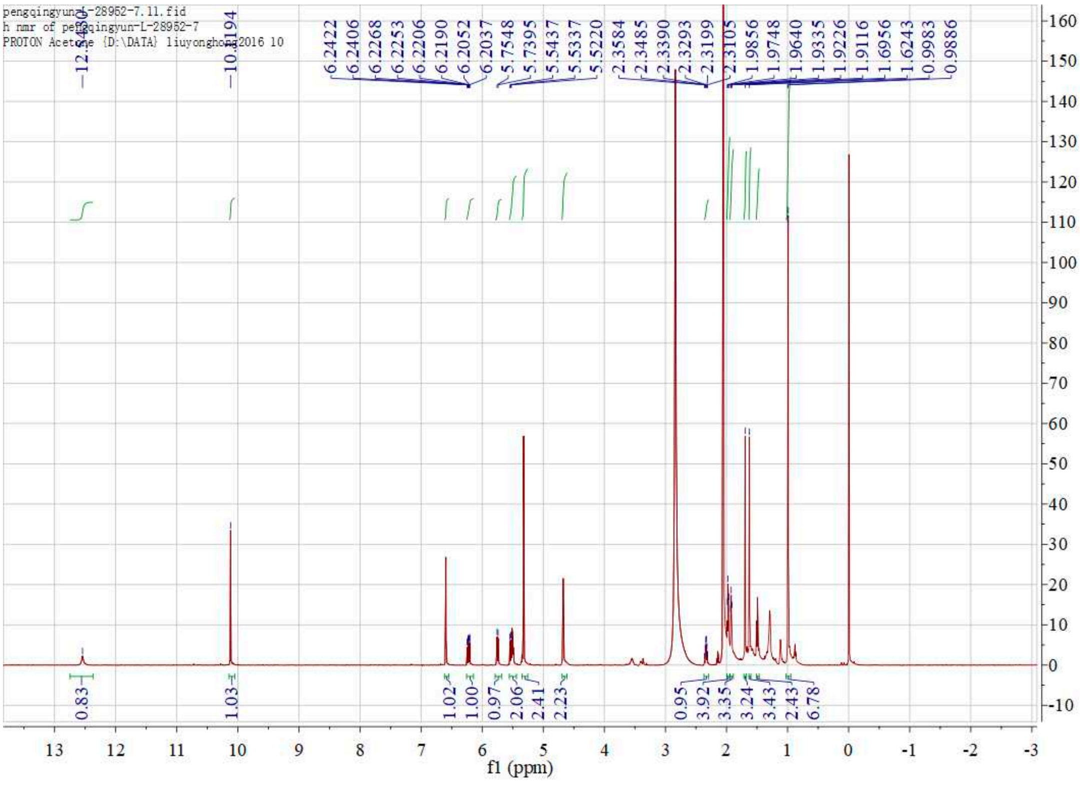

Figure S23 ^{1}H-NMR spectrum of compound **4**

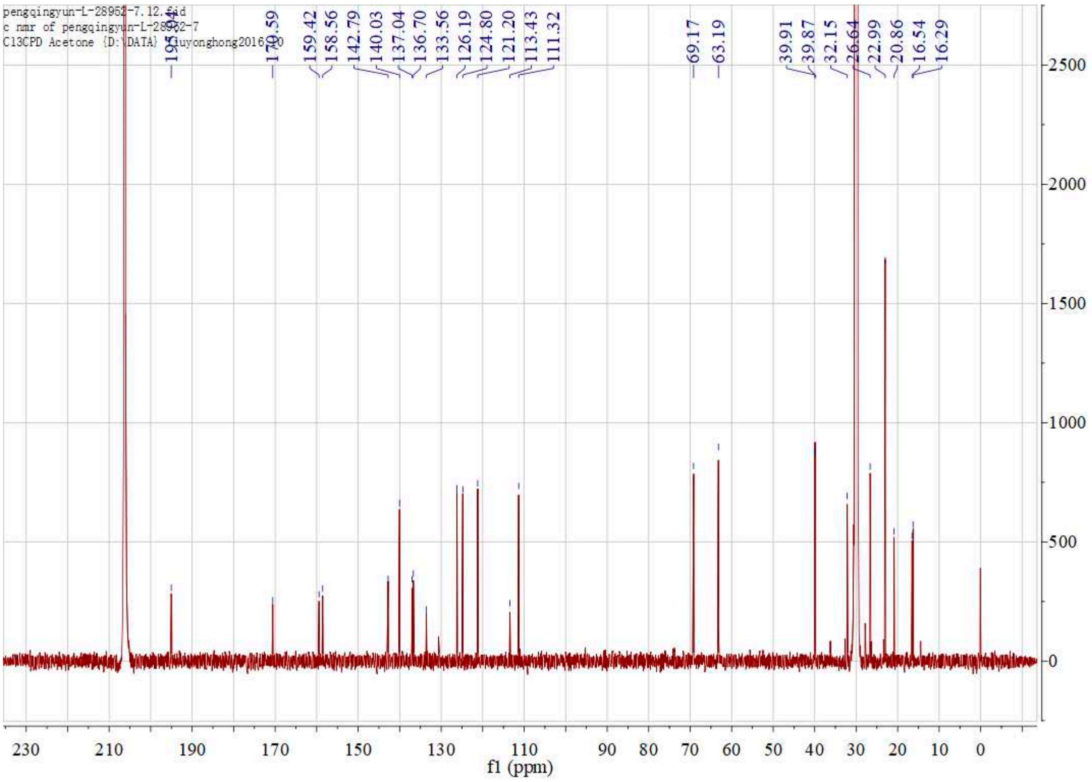

Figure S24 ^{13}C-NMR spectrum of compound **4**

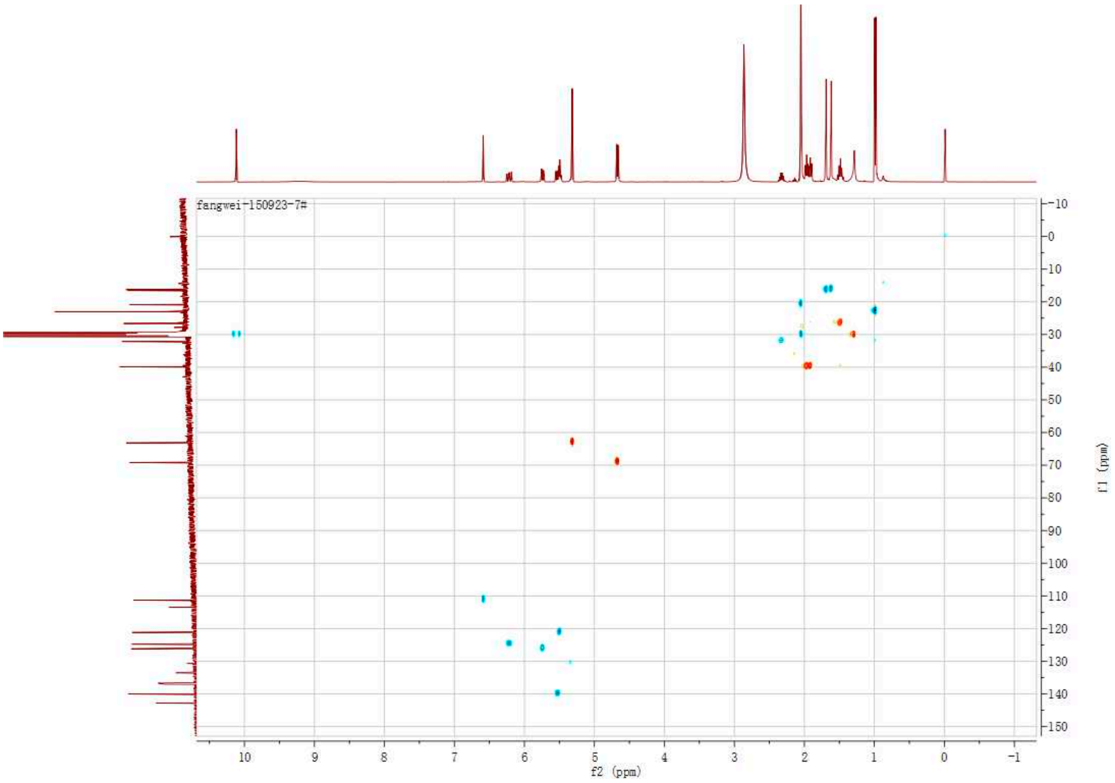

Figure S25 ^{1}H-^{13}C HSQC spectrum of compound **4**

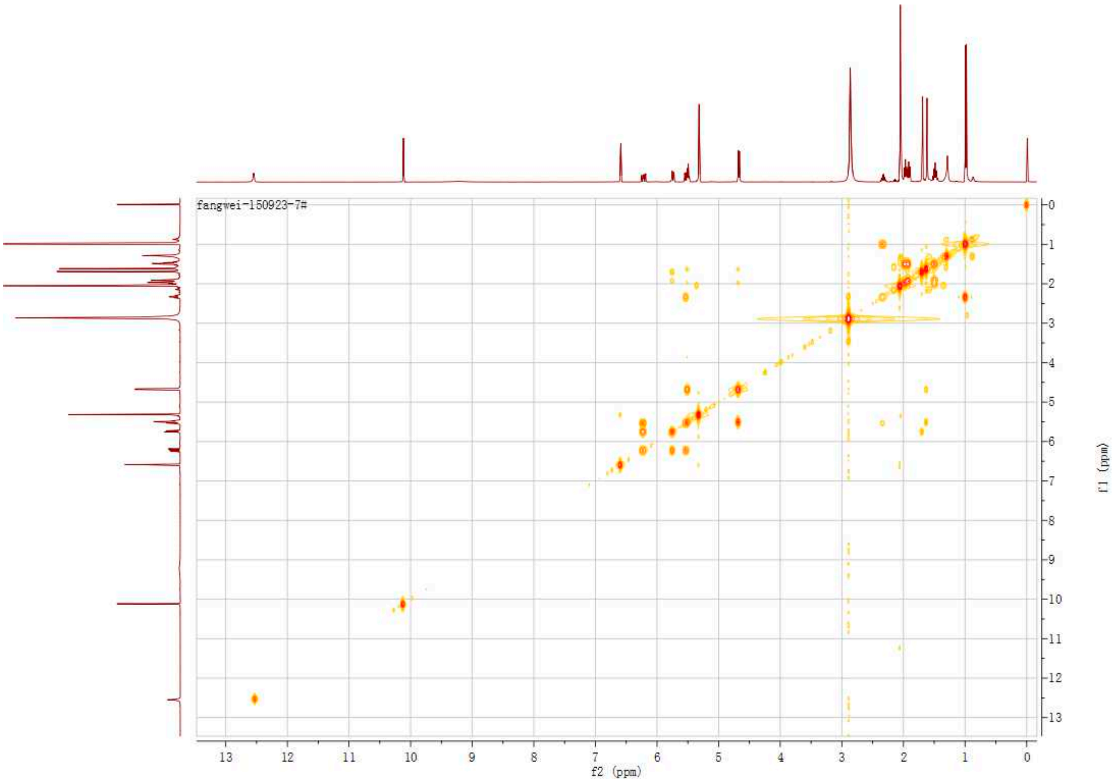

Figure S26 ^1H-^1H COSY spectrum of compound **4**

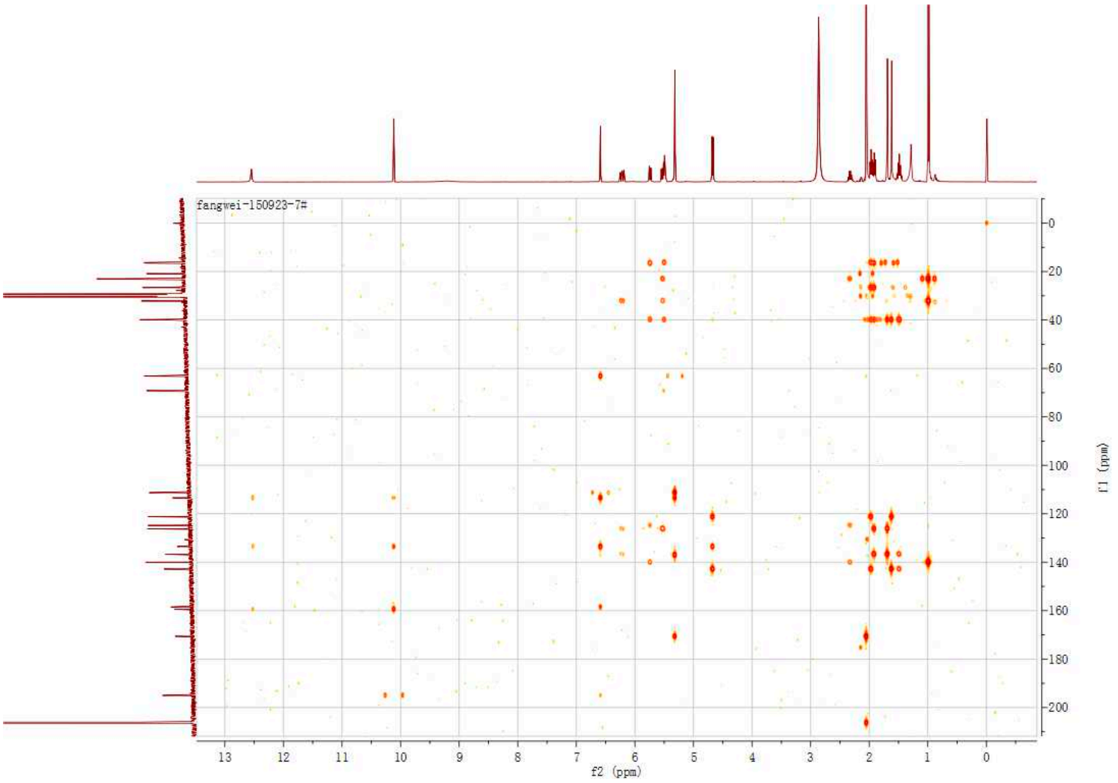

Figure S27 ^1H-^{13}C HMBC spectrum of compound **4**

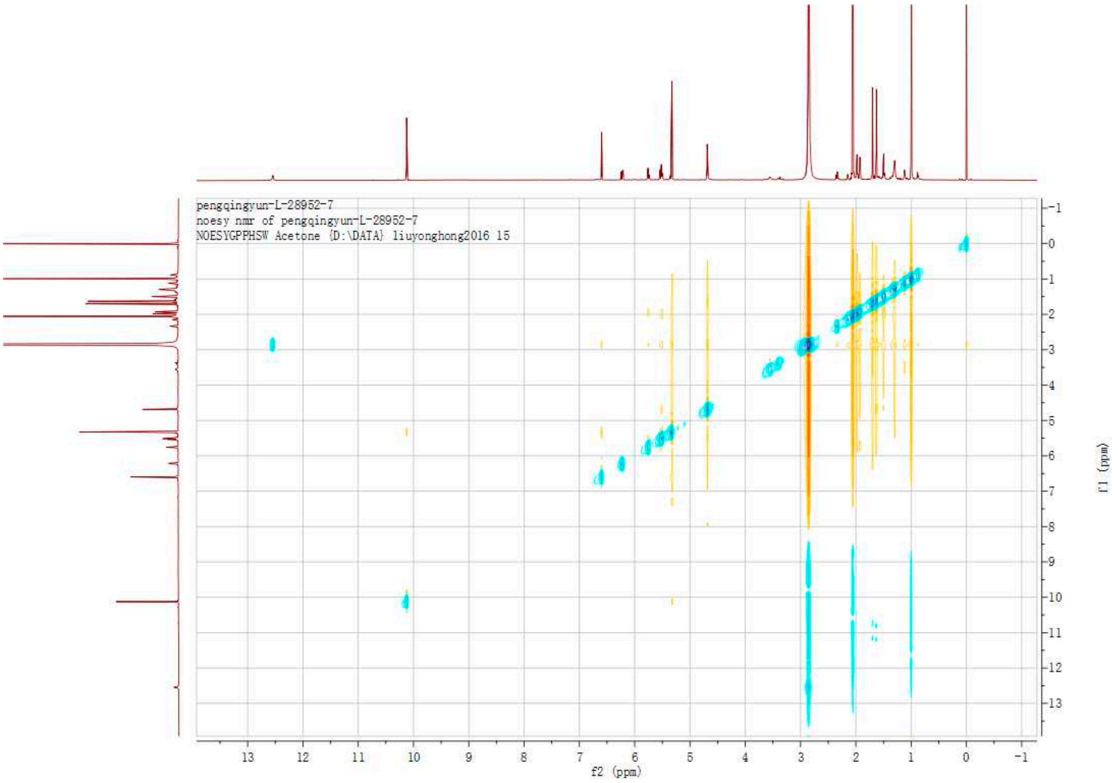

Figure S28 ^{1}H-^{1}H NOESY spectrum of compound **4**

Mass Spectrum SmartFormula Report

Analysis Info

Analysis Name: D:\Data\MS\data\202203\caijian_L-28952-9_pos_63_01_12826.d
Method: LC_Direct Infusion_pos_70-500mz.m
Sample Name: caijian_L-28952-9_pos
Comment

Acquisition Date: 3/16/2022 3:22:13 PM
Operator: SCSIO
Instrument: maXis 255552.00029

Acquisition Parameter

Source Type	ESI	Ion Polarity	Positive	Set Nebulizer	0.4 Bar
Focus	Active	Set Capillary	4500 V	Set Dry Heater	180 °C
Scan Begin	70 m/z	Set End Plate Offset	-500 V	Set Dry Gas	4.0 l/min
Scan End	1500 m/z	Set Charging Voltage	0 V	Set Divert Valve	Waste
		Set Corona	0 nA	Set APCI Heater	0 °C

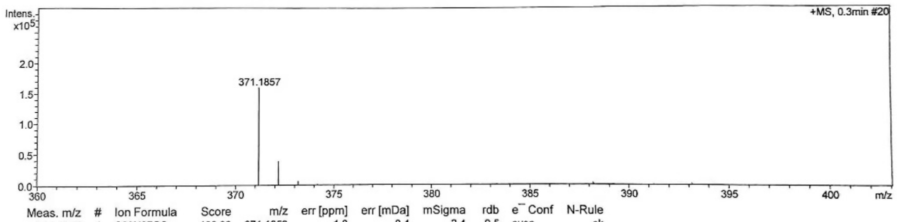

Meas. m/z	#	Ion Formula	Score	m/z	err [ppm]	err [mDa]	mSigma	rdb	e⁻ Conf	N-Rule
371.1857	1	C22H27O5	100.00	371.1853	1.0	0.4	2.4	9.5	even	ok
393.1688	1	C22H26NaO5	100.00	393.1672	-4.1	-1.6	13.9	9.5	even	ok
741.3641	1	C44H53O10	100.00	741.3633	1.0	0.7	23.6	18.5	even	ok
763.3431	1	C44H52NaO10	100.00	763.3453	-2.8	-2.2	35.9	18.5	even	ok

caijian_L-28952-9_pos_63_01_12826.d
Bruker Compass DataAnalysis 4.1 printed: 3/16/2022 3:27:53 PM by: SCSIO Page 1 of 1

Figure S29 Mass spectrum of compound **5** (determined as $C_{22}H_{26}O_5$ by HR-ESI-MS: *m*/*z* 371.1857, $[M+Na]^+$, cald for $C_{22}H_{27}O_5$, 371.1853)

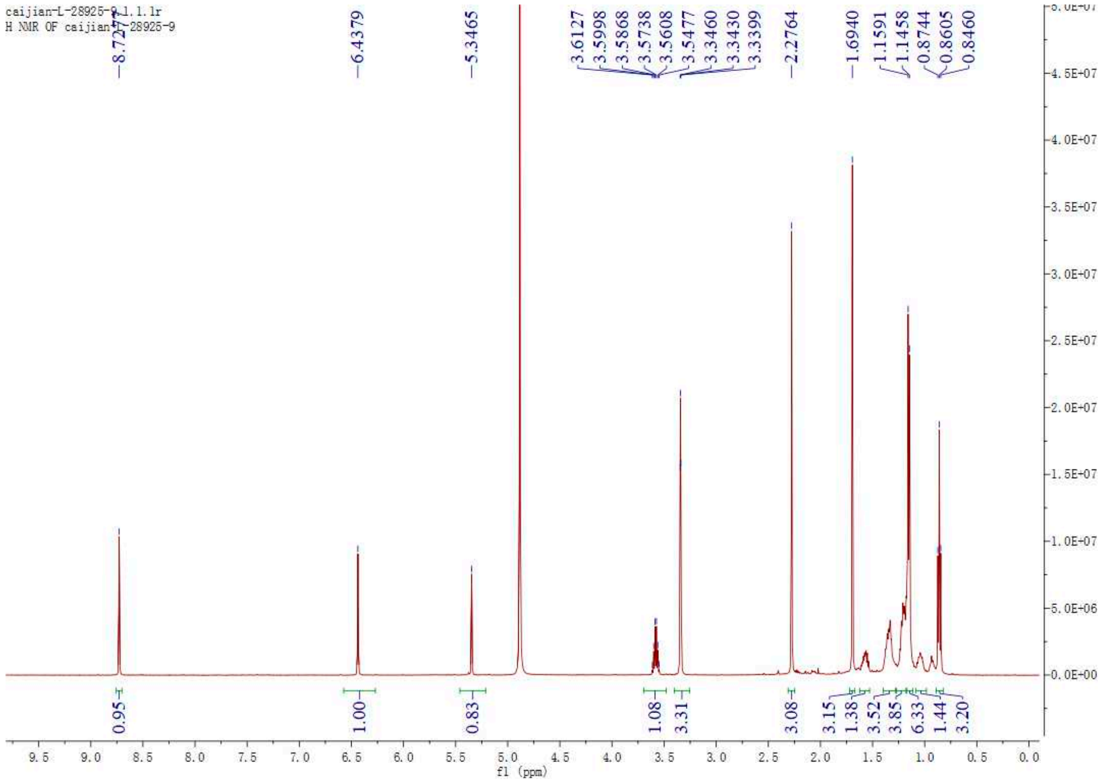

Figure S30 ^{1}H-NMR spectrum of compound **5**

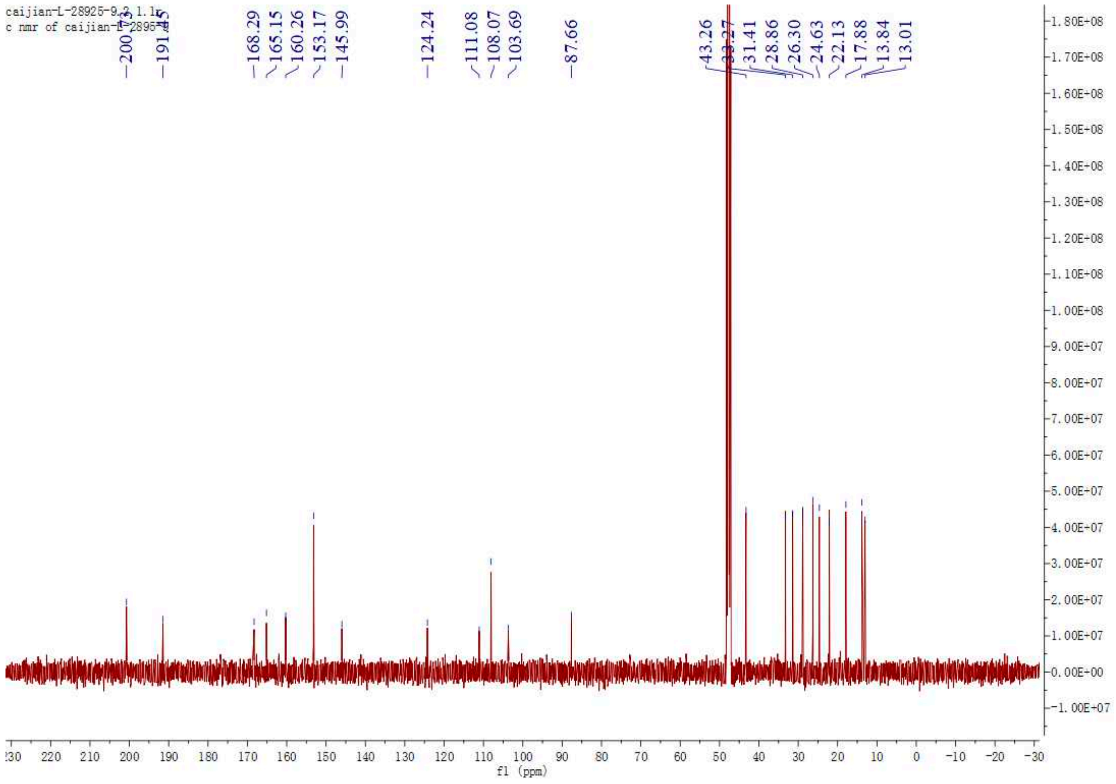

Figure S31 ^{13}C-NMR spectrum of compound **5**

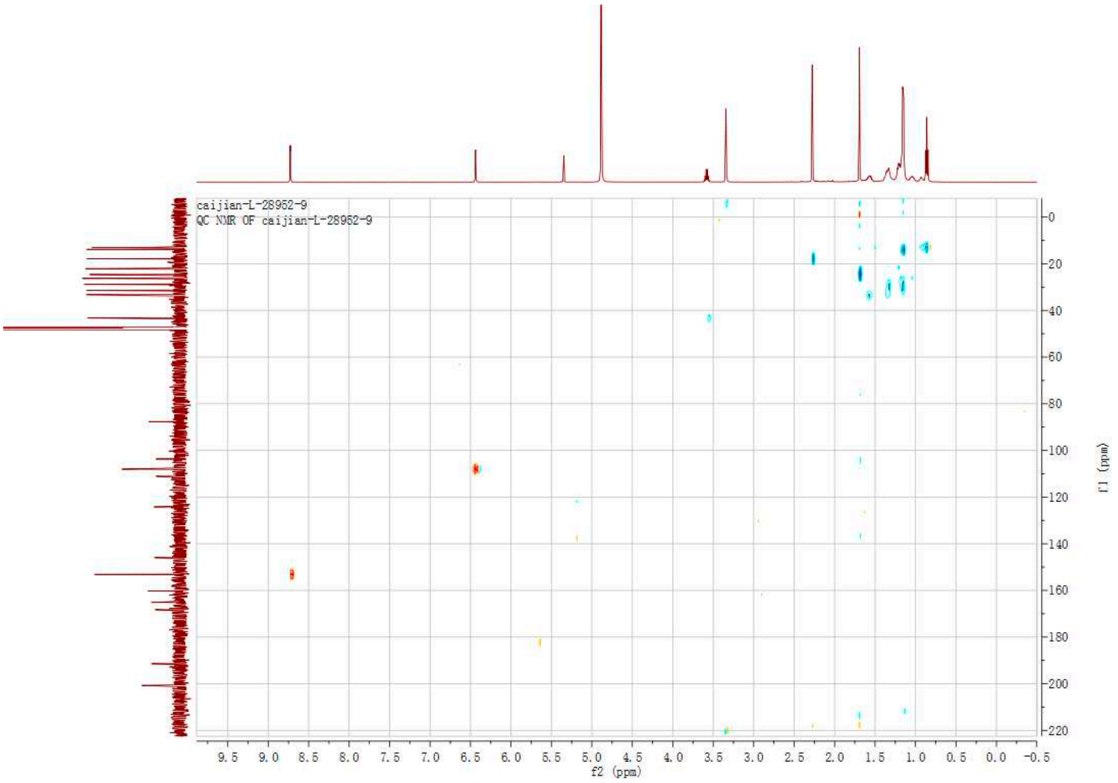

Figure S32 ^{1}H-^{13}C HSQC spectrum of compound **5**

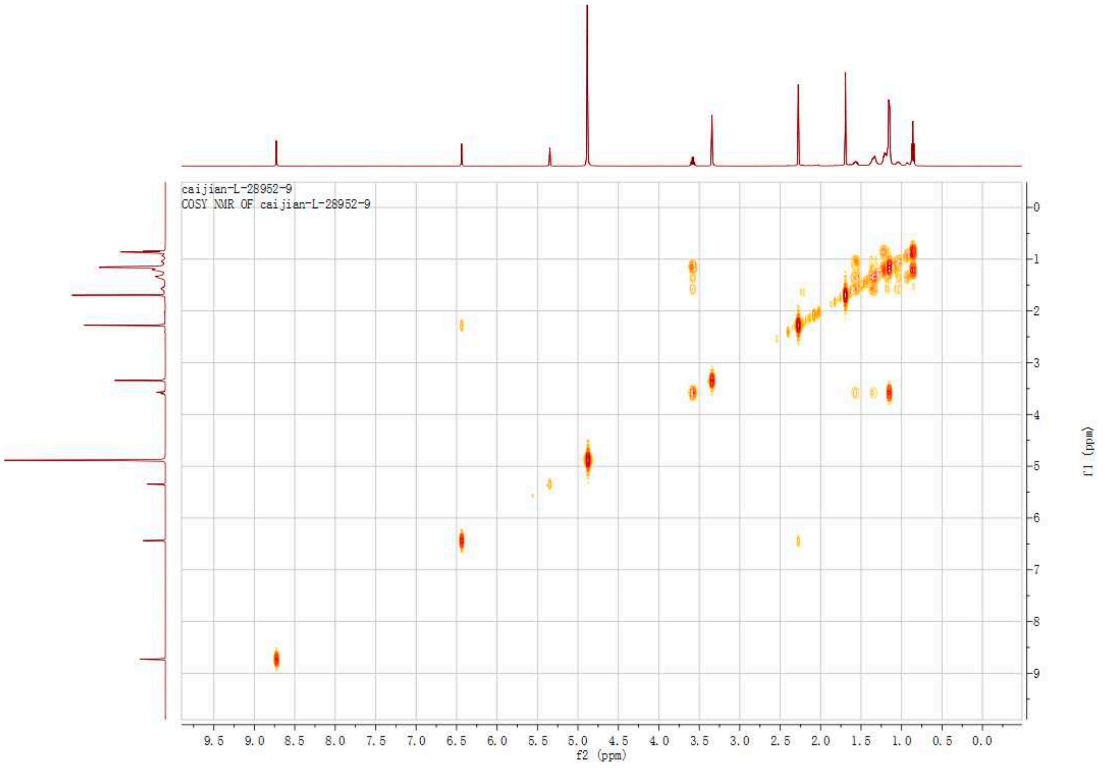

Figure S33 ^{1}H-^{1}H COSY spectrum of compound **5**

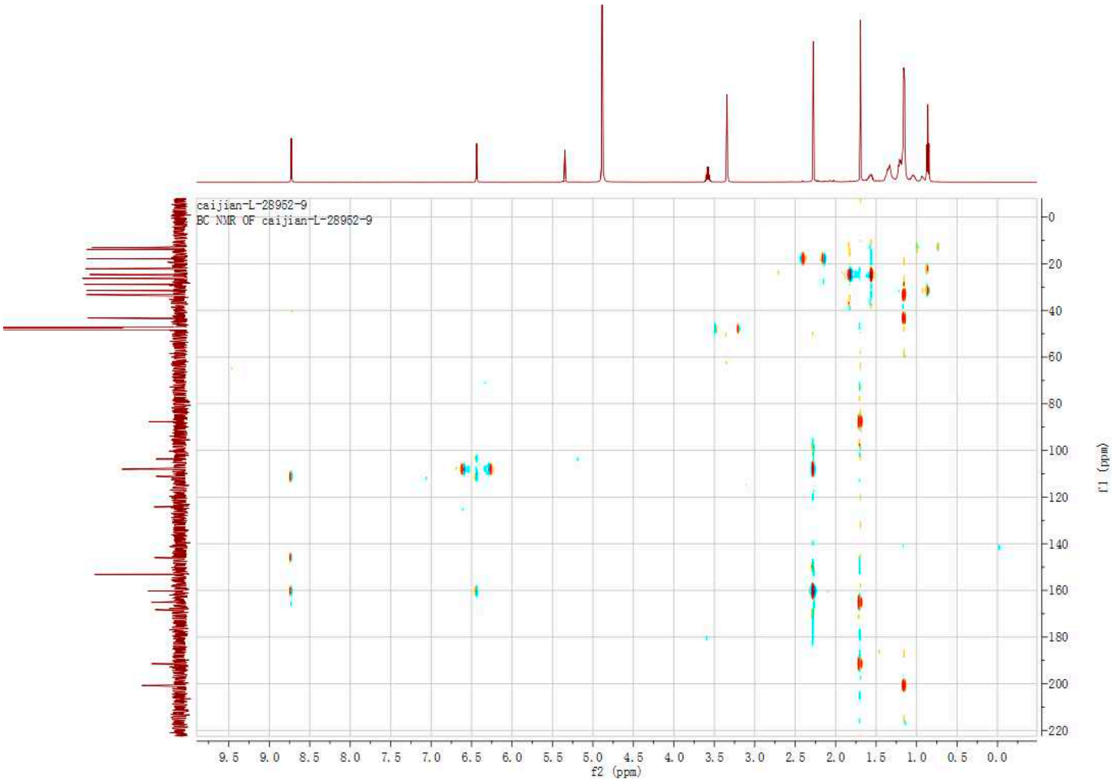

Figure S34 ^{1}H-^{13}C HMBC spectrum of compound **5**
